# Multidisciplinary Management of Low-velocity Nonmissile Penetrating Head Injuries

**DOI:** 10.7759/cureus.7388

**Published:** 2020-03-24

**Authors:** Michael Young, Matthew Putty, Megan M Finneran, Ryan Johnson, Keith Schaible, Hamad Farhat

**Affiliations:** 1 Neurological Surgery, Advocate Health Care, Oak Lawn, USA; 2 Neurological Surgery, Advocate Health Care, Normal, USA; 3 Neurological Surgery, Advocate BroMenn Medical Center, Normal, USA; 4 Neurological Surgery, Advocate Christ Medical Center, Oak Lawn, USA

**Keywords:** low velocity non-missile penetrating head injury, arterial injury, neuroendovascular management, endoscopic approach

## Abstract

Introduction

Penetrating head injuries (PHIs) can have diverse presentations and mechanisms; therefore, treatment methods have not been clearly outlined. Vascular injury is common and foreign body removal is often required. We present three cases to illustrate low-velocity nonmissile penetrating head injuries (NPHIs) and discuss a multidisciplinary approach.

Methods

We present a case series from our institution that illustrates the importance of multidisciplinary treatment of these injuries. All injuries are low- velocity NPHIs with separate mechanisms and anatomical locations.

Results

Multidisciplinary management involving neurosurgery, otolaryngology, and neuroendovascular surgery is represented in our case series with all patients having good clinical outcomes. Our first case is a 34-year-old male who presented neurologically intact after a stabbing in the left temporal region with concerns for external carotid artery injury and maxillary sinus injury. Our second case is a 37-year-old male who presented with a self-inflicted nail gun injury that penetrated the right temporal bone, right temporal lobe, bilateral sphenoid sinus, and left petrous carotid canal with concerns of petrous internal carotid injury. Our third case is a 31-year-old male who presented after an accidental nail gun injury that penetrated through the oral cavity, hard palate, and left sphenoid sinus and ending in the left cavernous sinus with concerns of cavernous internal carotid injury.

Conclusion

Careful consideration must be taken when evaluating low-velocity NPHIs. Particular attention must be given when an associated vascular injury is suspected. Our case series highlights the importance of a multidisciplinary approach in achieving good clinical outcomes in PHIs.

## Introduction

Penetrating head injuries (PHIs) are a severe subtype of traumatic brain injuries (TBIs). The pathophysiology and management of missile PHIs, most often caused by a blast phenomenon from ammunition such as guns, have been established and studied in the military and civilian populations [1]. Nonmissile penetrating head injuries (NPHIs), on the other hand, are relatively rare; and theories about their management are mostly controversial. They are defined to have an impact velocity of less than 100 meters per second [1-5]. Although less destructive than missile PHIs, significant damage can occur when vital structures are impacted. The first reported case of NPHI dates back to 1806 [6]. Reports of NPHIs have included such objects as knives, pencils, pens, nails, toothbrushes, wooden sticks, screwdrivers, keys, arrows, and scissors, but no clear management has been defined [6-7]. We present our experience with three cases of NPHIs and review the literature on injuries of this nature. Our primary objective is to highlight the importance of a multidisciplinary approach in addressing NPHIs to create an optimal outcome.

## Materials and methods

We present a case series from our institution that illustrates the importance of multidisciplinary treatment of NPHIs. All injuries we present are low-velocity NPHIs with separate mechanisms and anatomical locations. We discuss the relevant literature and similar cases at other institutions.

## Results

Case 1

**Figure 1:**
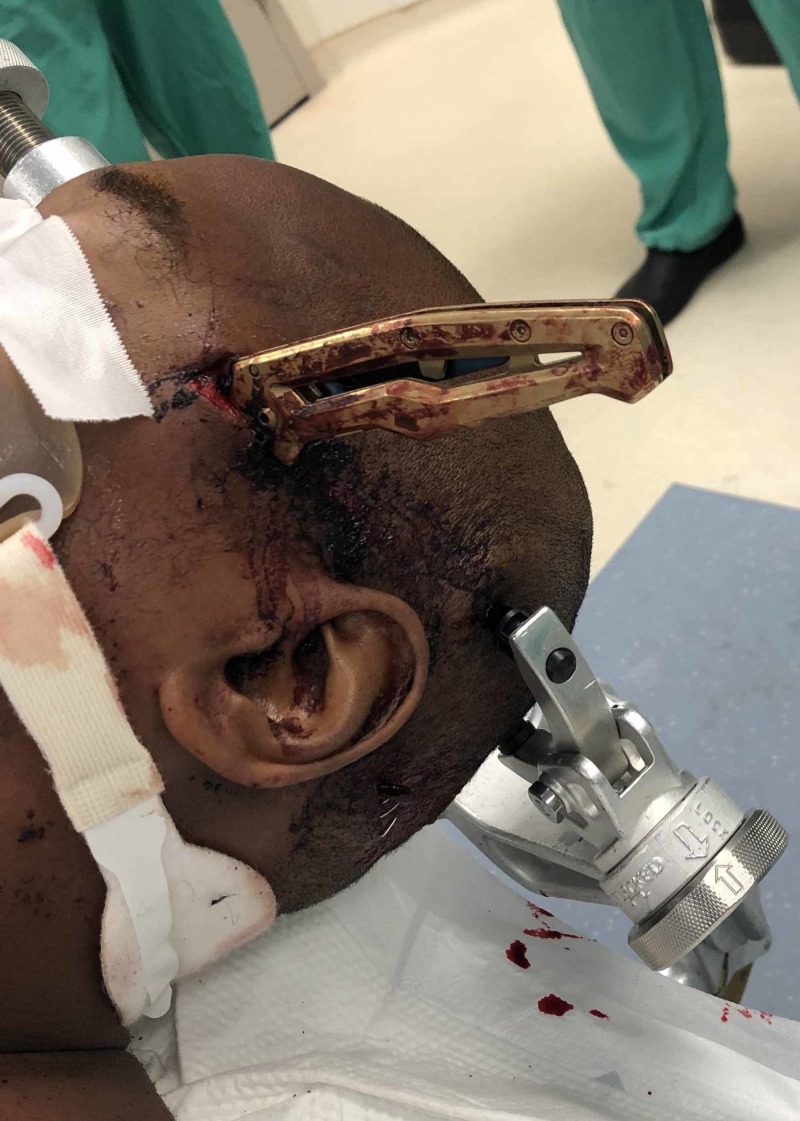
Knife penetrating the left temporal scalp and skull

**Figure 2:**
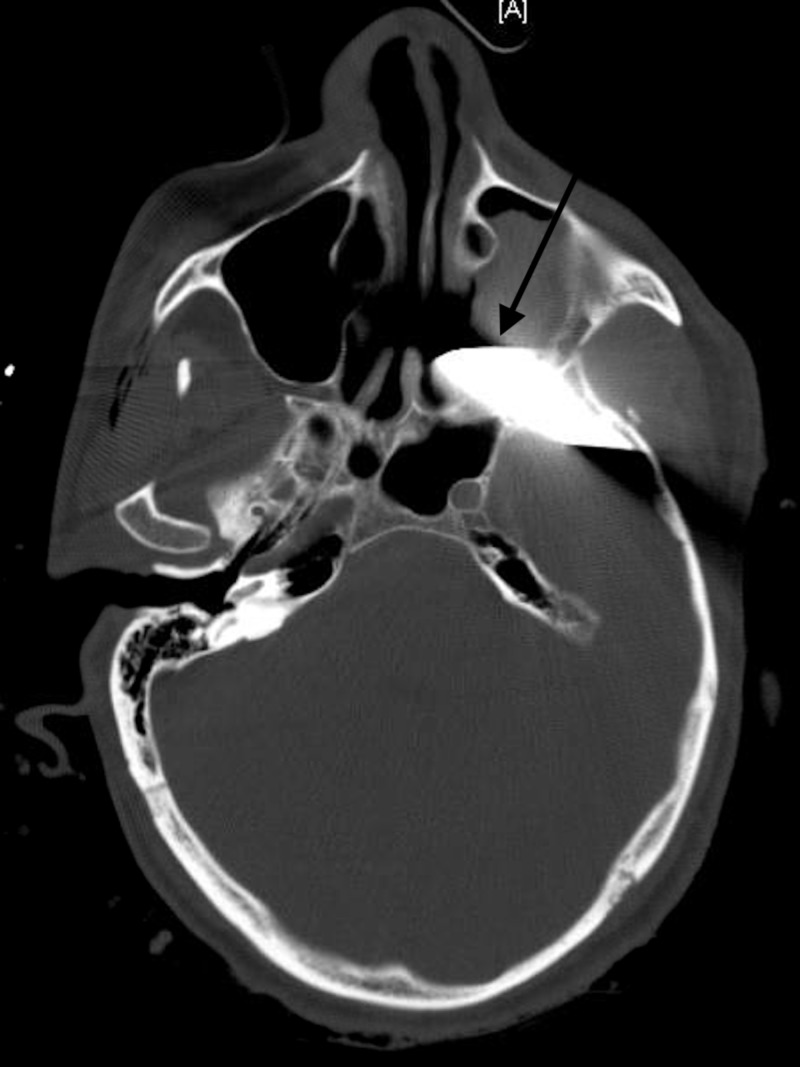
CT of the head The image shows knife penetrating the left temporal bone, left sphenoid sinus, left pterygopalatine fossa, and left maxillary sinus (black arrow) CT: computed tomography

A 34-year-old male presented to the trauma bay neurologically intact after sustaining a stabbing to the left temporal region (Figure 1). A CT of the head showed that the knife had penetrated the left temporal bone, left sphenoid bone, left pterygopalatine fossa, and left maxillary sinus (Figure 2).

**Figure 3:**
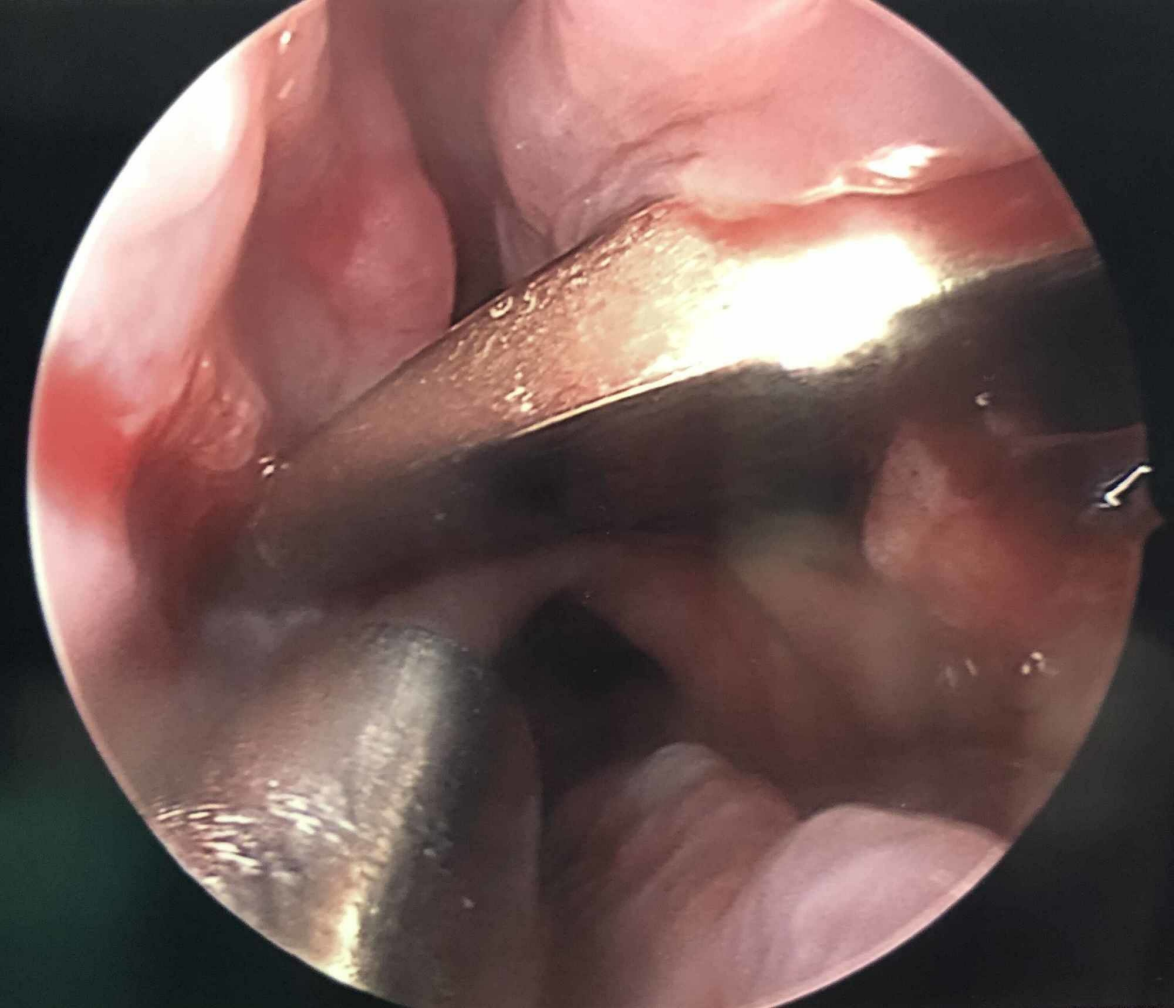
Endoscopic visualization of the knife in the maxillary sinus

A computed tomography angiogram (CTA) showed no obvious vascular injury, but the process was limited due to streak artifact. Since the knife penetrated the maxillary sinus and there was a concern for a vascular injury, the patient was planned for multidisciplinary treatment with neurosurgery, otolaryngology, and neuroendovascular surgery. The patient was taken to the operating room for the planned removal of the knife under endoscopic visualization and wound debridement. A small incision was made around the knife and the endoscope was advanced into the maxillary sinus with immediate visualization of the knife (Figure 3).

**Figure 4:**
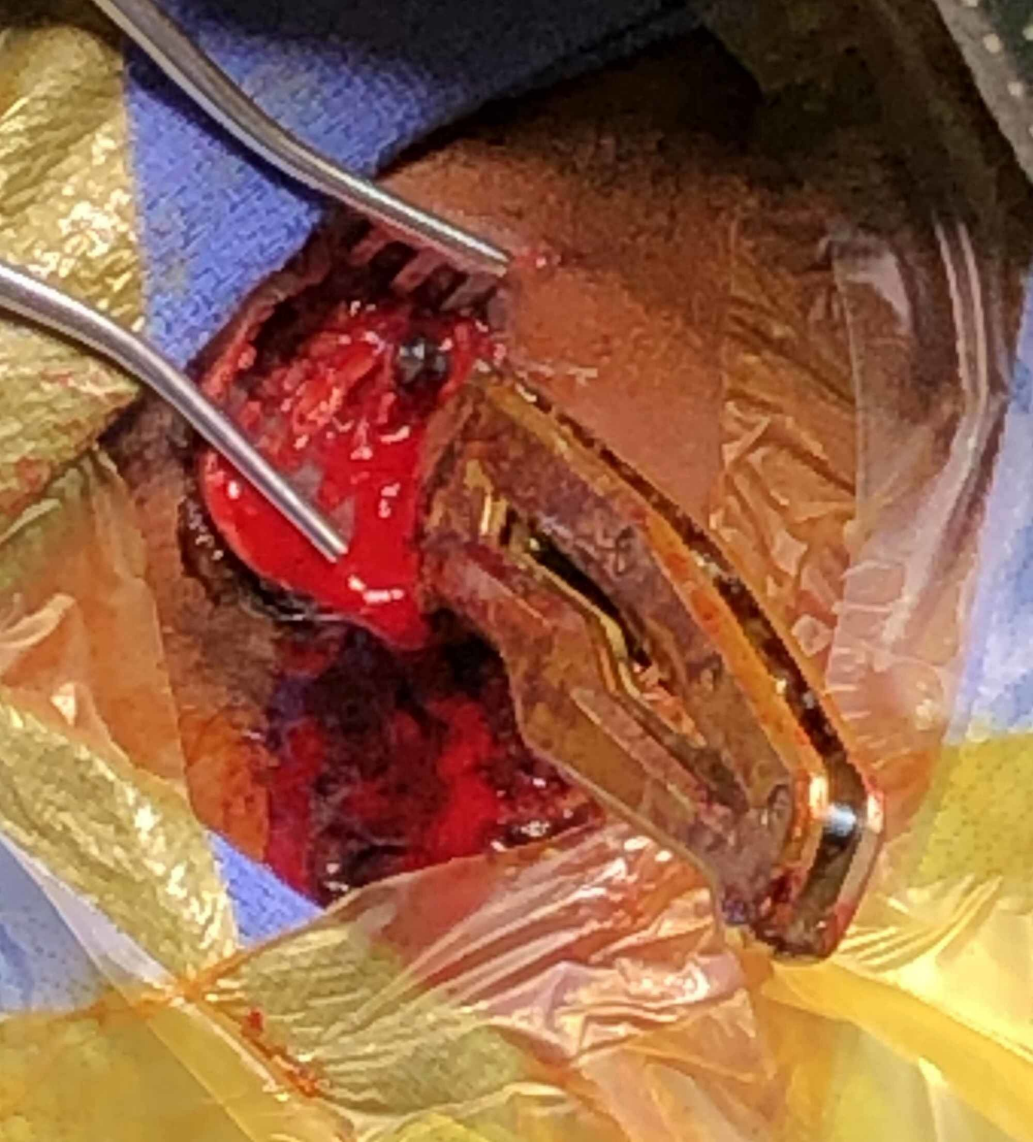
Intraoperative removal of the knife under direct visualization

Under direct visualization, the knife was removed with minimal bleeding (Figure 4).

**Figure 5:**
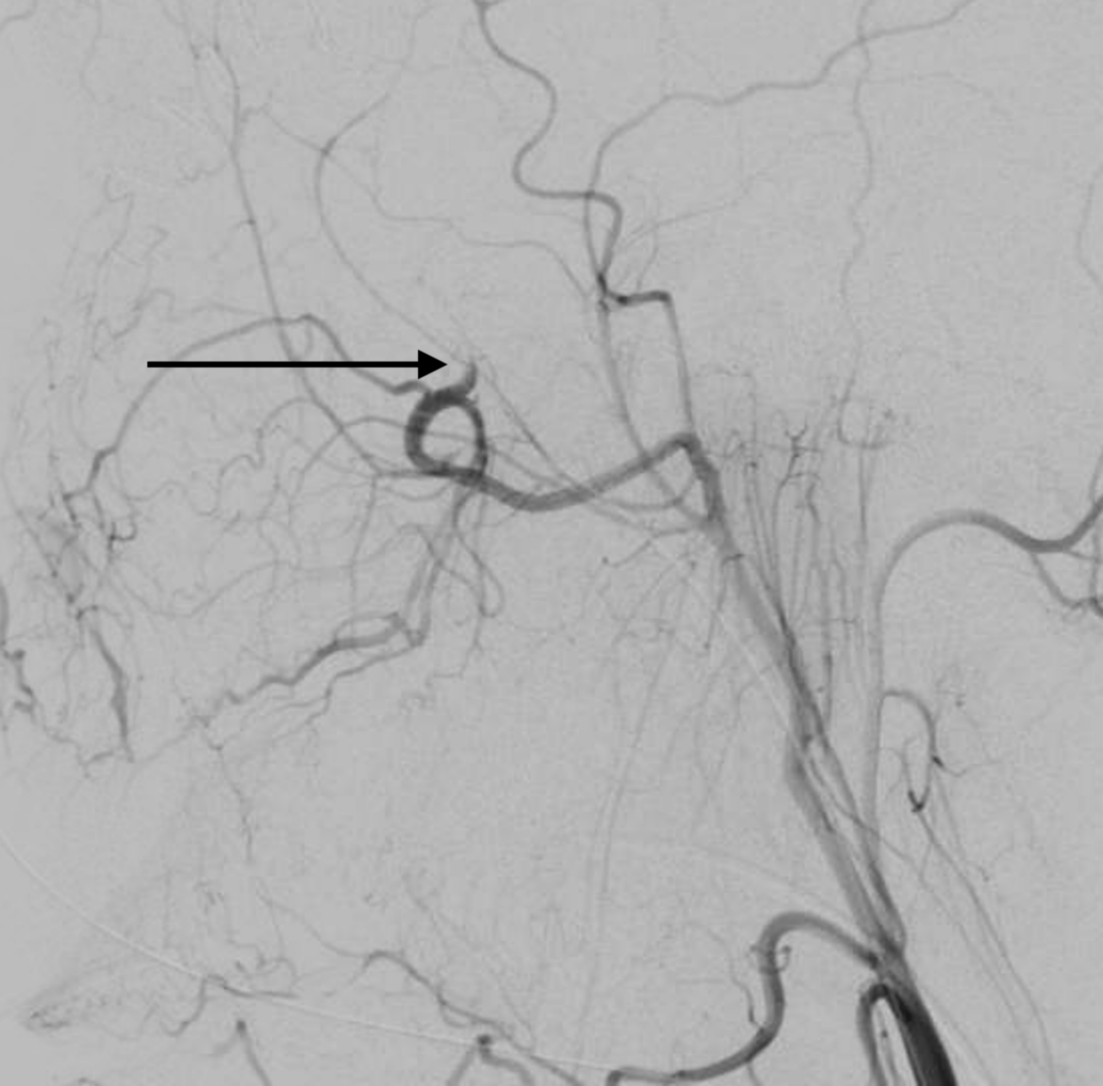
Lateral projection DSA The image shows the left distal internal maxillary artery and proximal middle meningeal artery injury (black arrow) DSA: digital subtraction angiography

The wound was then debrided of bone fragments and copiously irrigated. The patient was taken immediately to the endovascular suite where digital subtraction angiography (DSA) showed a left distal internal maxillary artery and proximal middle meningeal artery injury (Figure 5). 

**Figure 6:**
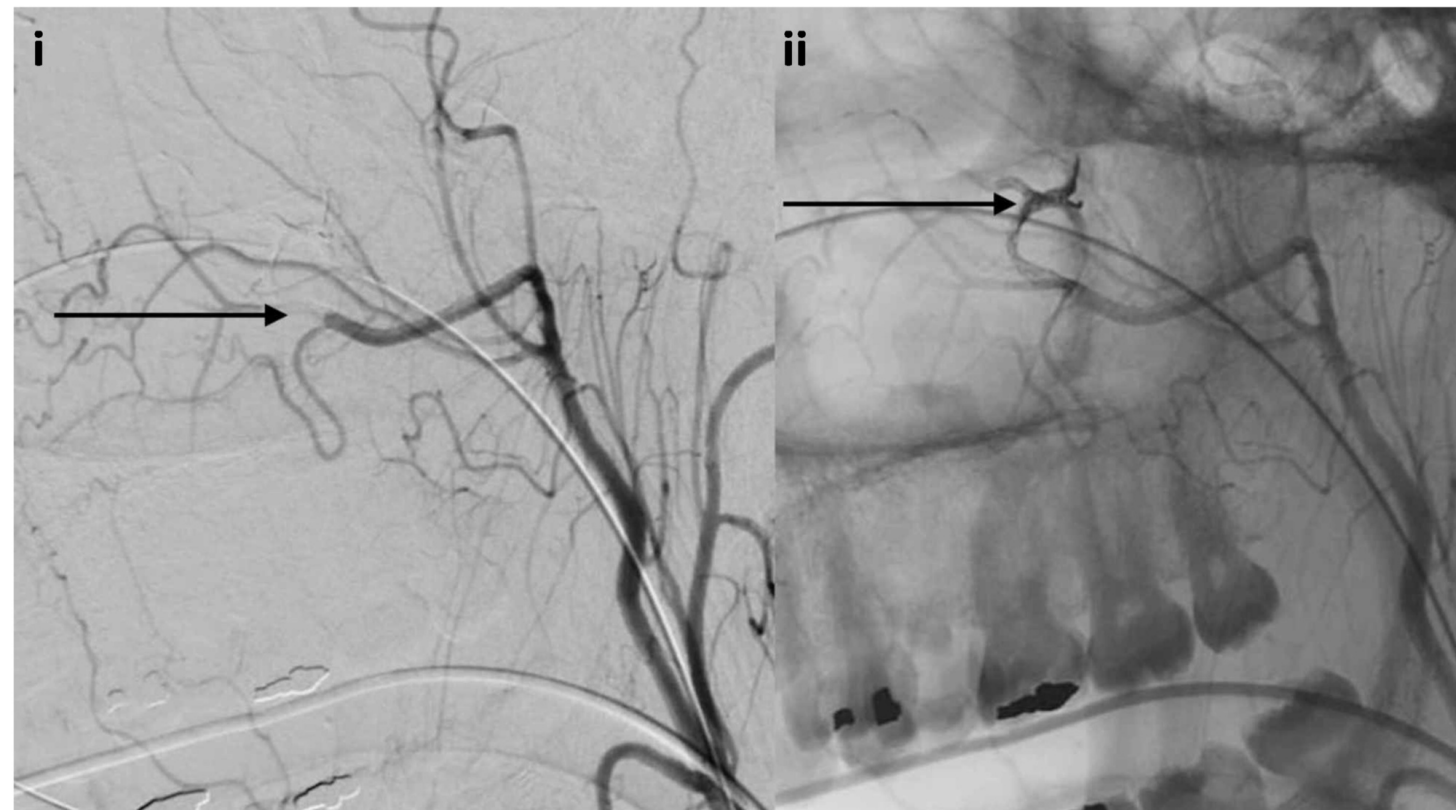
Lateral projection DSA and unsubtracted lateral projection angiogram i) Lateral projection DSA demonstrating Onyx embolization of left distal internal maxillary artery and proximal middle meningeal artery injury (black arrow); ii) Unsubtracted lateral projection angiogram showing Onyx embolic material (black arrow) DSA: digital subtraction angiography

The left distal internal maxillary artery and proximal middle meningeal artery were embolized with Onyx (ev3, Irvine, CA) due to active bleeding (Figure 6).

**Figure 7:**
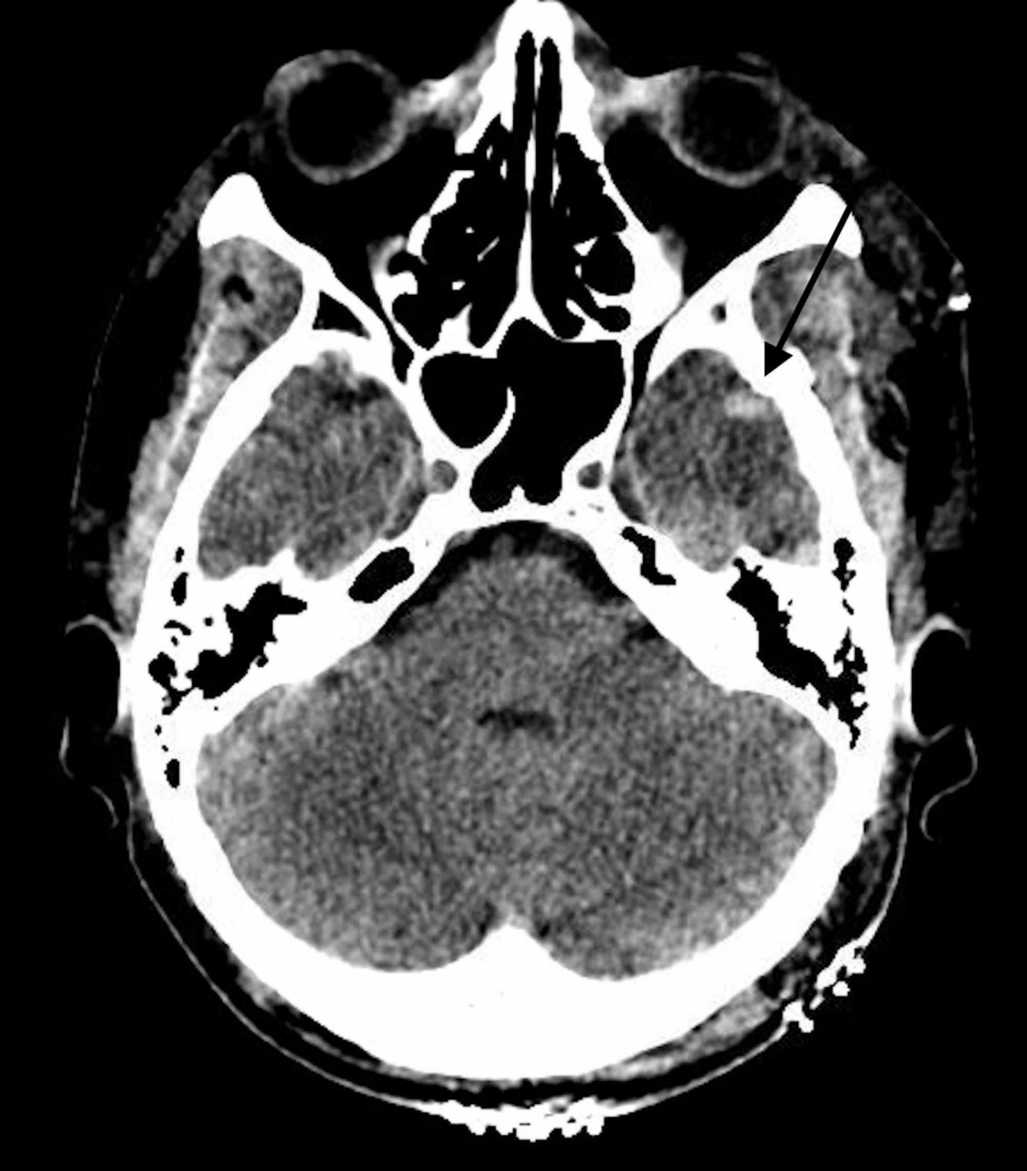
Postoperative CT of the head The image shows small left temporal lobe intracerebral hemorrhage (black arrow) CT: computed tomography

The postoperative head CT showed a very small left temporal lobe hemorrhage with a small amount of pneumocephalus without any hydrocephalus (Figure 7).

The patient was extubated on post-injury day one without any cranial nerve palsies or other neurologic deficits and was discharged on postoperative day two.

Case 2

**Figure 8:**
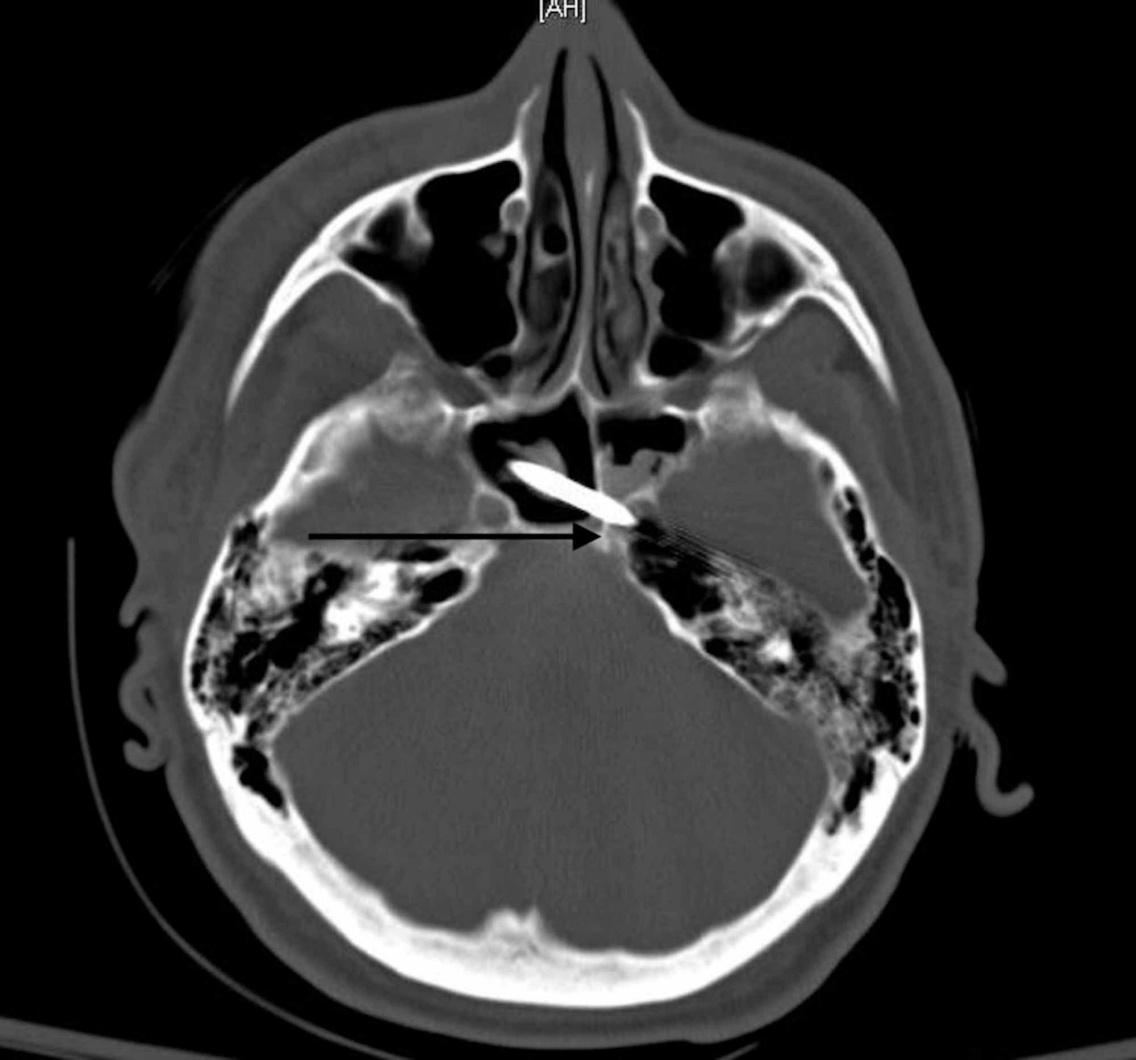
CT of the head The image shows the nail penetrating left petrous carotid canal (black arrow) CT: computed tomography

A 37-year-old male presented with a self-inflicted nail gun injury that had penetrated the right temporal bone, right temporal lobe, bilateral sphenoid sinus, and left petrous carotid canal (Figure 8).

**Figure 9:**
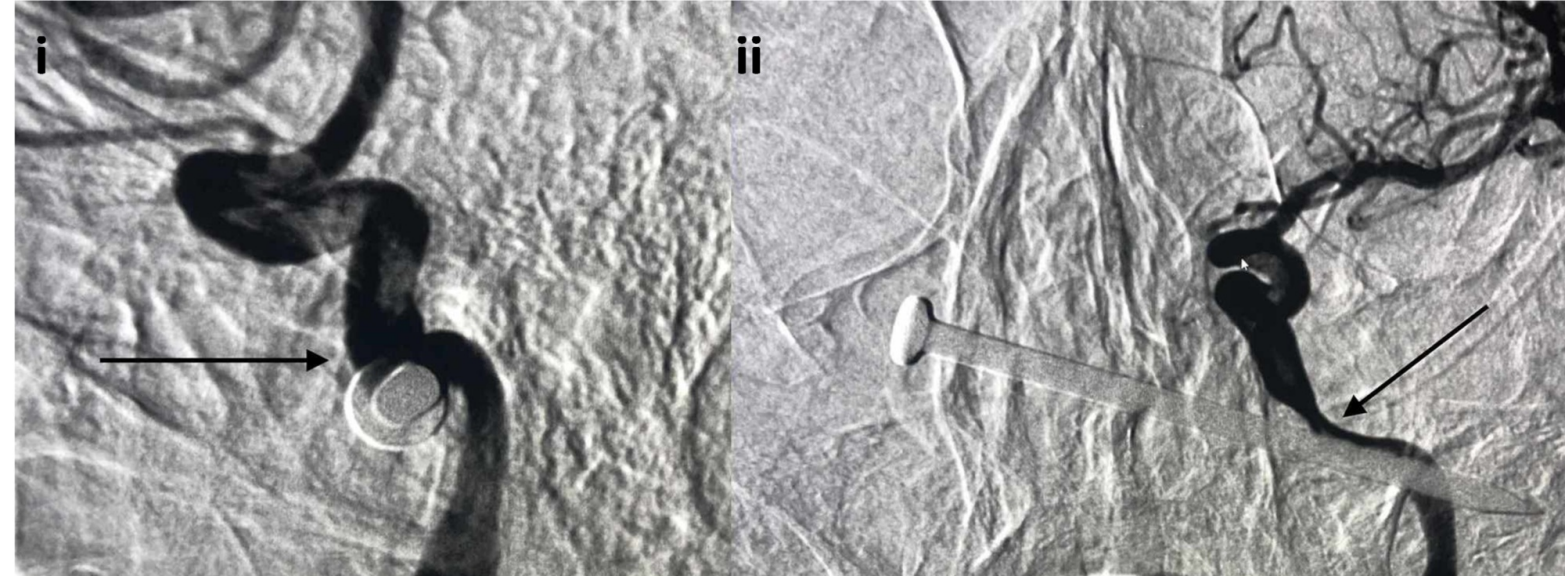
Initial lateral and AP projection DSA i) Lateral projection DSA demonstrating vasospasm of petrous internal carotid artery but with no evidence of traumatic pseudoaneurysm (black arrow); ii) AP projection DSA demonstrating petrous internal carotid artery but with no evidence of traumatic pseudoaneurysm (black arrow) DSA: digital subtraction angiography; AP: anterior-posterior

He presented with a right sixth cranial nerve palsy but was otherwise neurologically intact. Initial CTA and DSA demonstrated narrowing of the petrous internal carotid artery, but there was no evidence of pseudoaneurysm or carotid artery injury (Figure 9).

**Figure 10:**
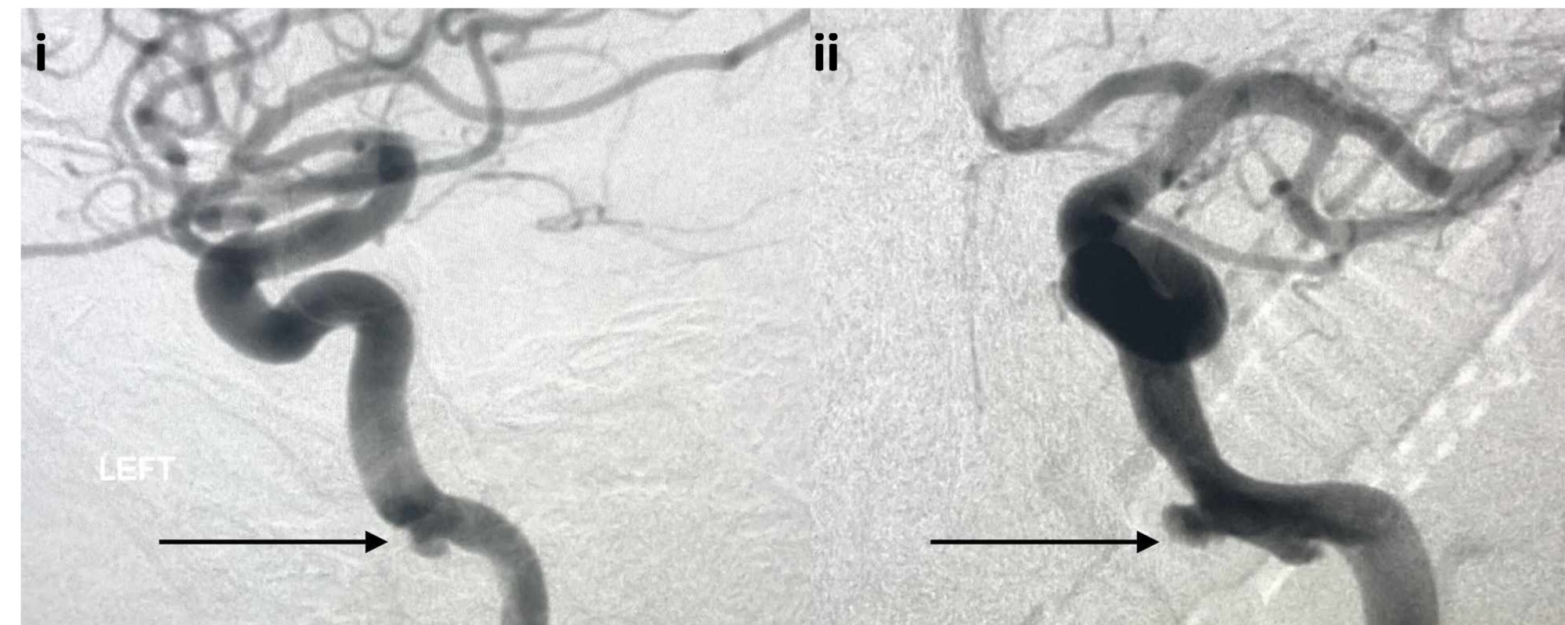
Follow-up lateral and AP projection DSA i) Lateral projection DSA demonstrating traumatic pseudoaneurysm of the left petrous internal carotid artery (black arrow); ii) AP projection DSA demonstrating traumatic pseudoaneurysm of the left petrous internal carotid artery (black arrow) DSA: digital subtraction angiography; AP: anterior-posterior

The patient was taken to the operating room for a combined right temporal craniotomy and transsphenoidal endoscopic approach to visualize the nail. The craniotomy extended around the nail and a depressed skull fracture, which was elevated. The nail was dissected down towards the skull base and, at that time, the endoscope was advanced into the sphenoid sinus with direct visualization of the nail crossing the sphenoid sinus. The nail was able to be removed under direct visualization; however, upon removal, there was brisk arterial blood filling the sphenoid sinus. The sphenoid sinus was densely packed with Surgicel (Ethicon, Somerville, NJ) and abdominal fat graft. One week later, the patient was taken to the endovascular suite. DSA showed a left petrous internal carotid artery injury with pseudoaneurysm (Figure 10).

**Figure 11:**
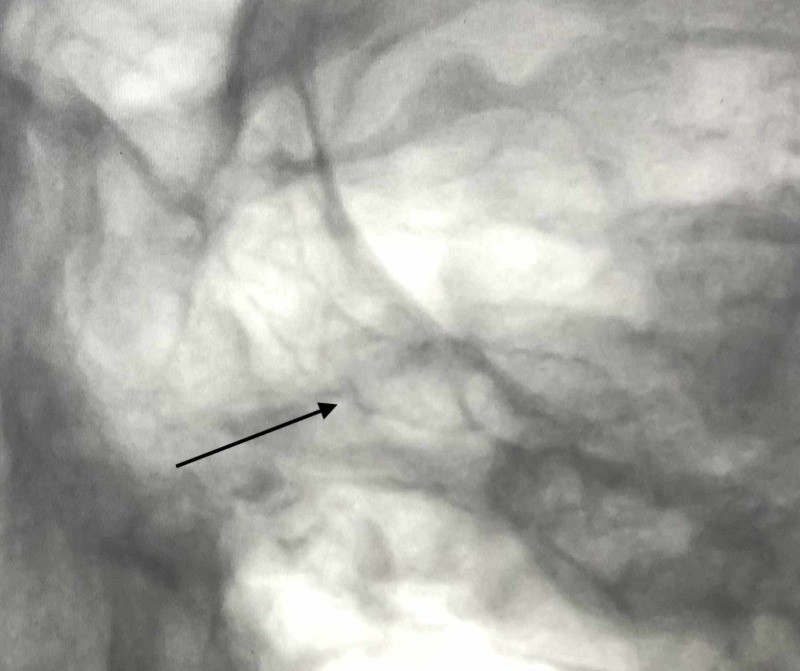
Unsubtracted lateral projection angiogram The image shows the successful deployment of the PED within the left petrous internal carotid artery (black arrow) PED: Pipeline Embolization Device

This was treated with Pipeline Embolization Device (PED; Medtronic, Irvine, CA) across the petrous internal carotid injury and pseudoaneurysm (Figure 11).

**Figure 12:**
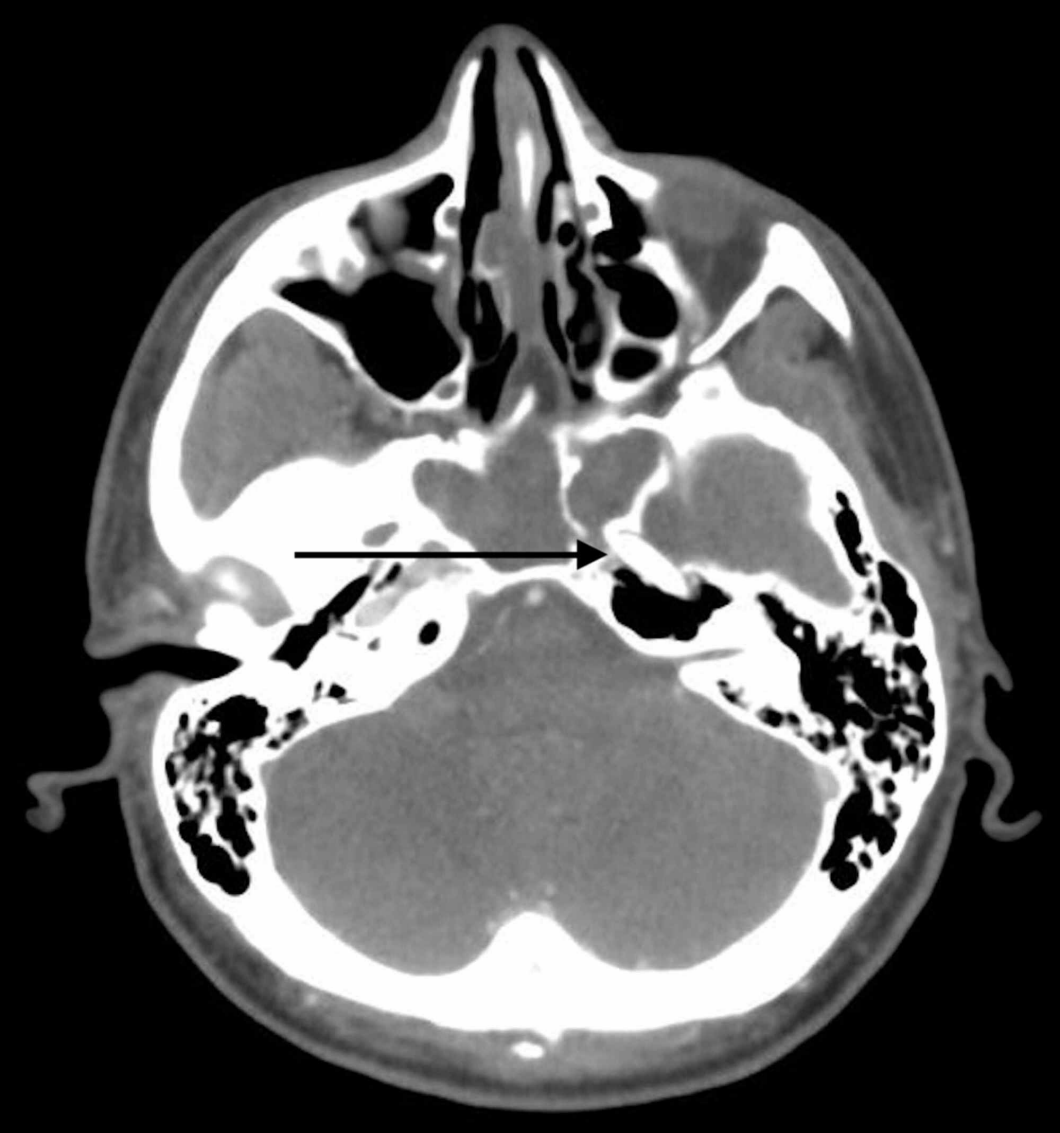
Follow-up CTA one week later The image shows the resolution of the left petrous carotid artery pseudoaneurysm (black arrow) CTA: computed tomography angiogram

A follow-up CTA one week later showed the resolution of the left petrous carotid artery pseudoaneurysm (Figure 12).

The patient was discharged to the inpatient psychiatric facility with improvement in his 6th cranial nerve palsy and he remained neurologically intact.

Case 3

**Figure 13:**
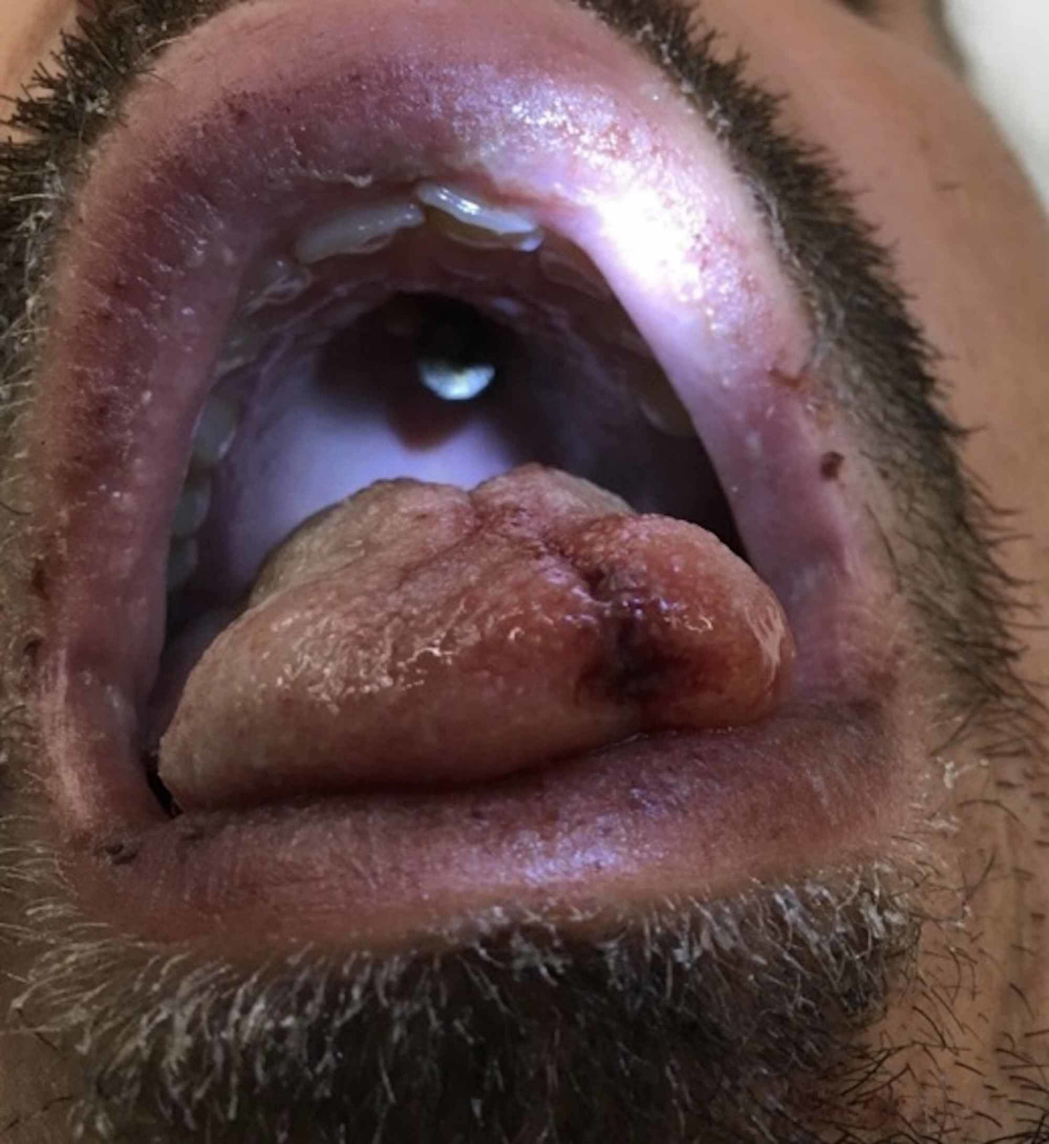
Nail penetrating the hard palate

 A 31-year-old male presented after an accidental nail gun injury that penetrated through the oral cavity, hard palate, and left sphenoid sinus, and ended in the left cavernous sinus (Figure 13).

**Figure 14:**
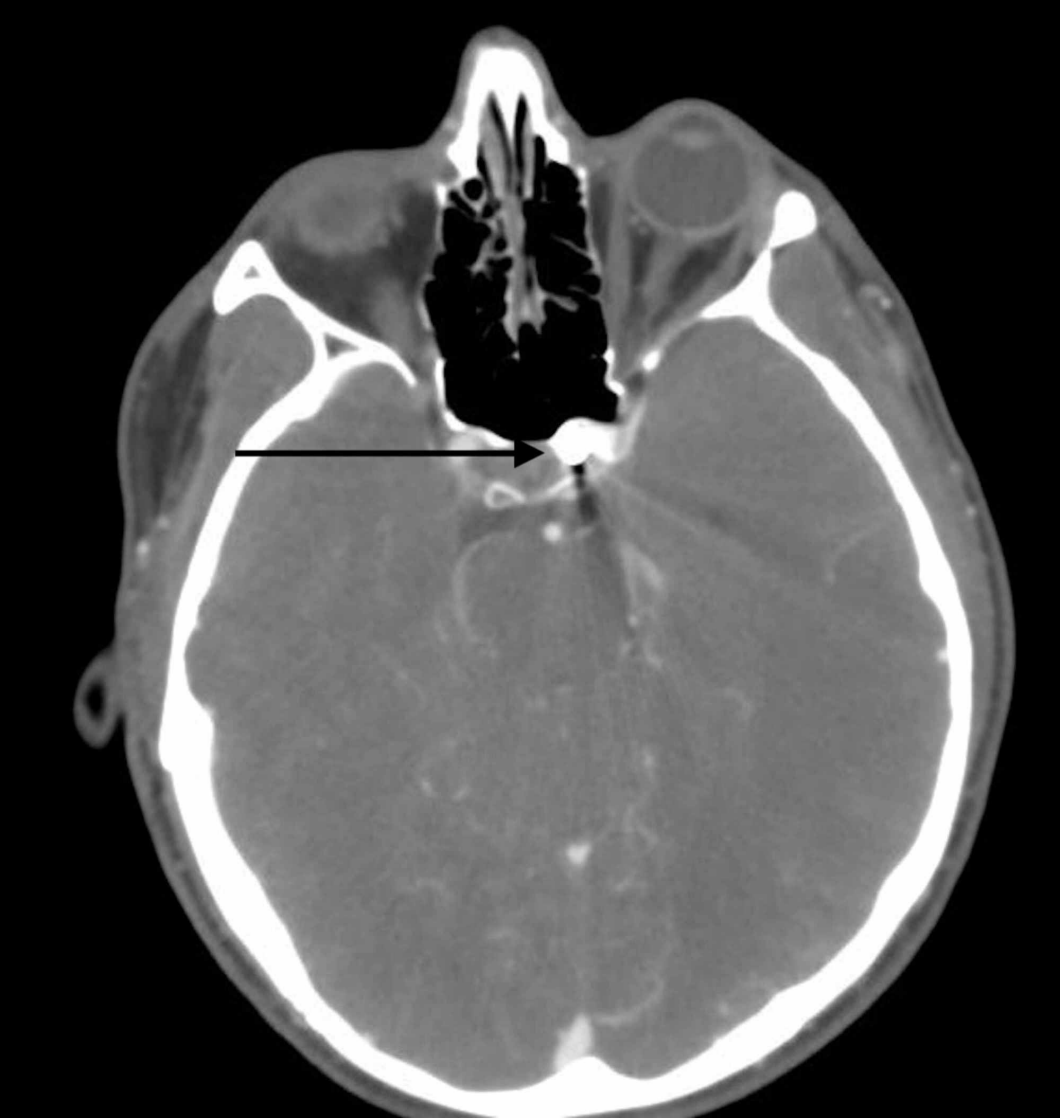
CTA of the injury The image shows the nail traversing the left internal carotid artery in the cavernous sinus in the carotid siphon region (black arrow) CTA: computed tomography angiogram

The patient presented neurologically intact and hemodynamically stable. A CTA revealed the nail traversing the left internal carotid artery in the cavernous sinus carotid siphon region (Figure 14).

**Figure 15:**
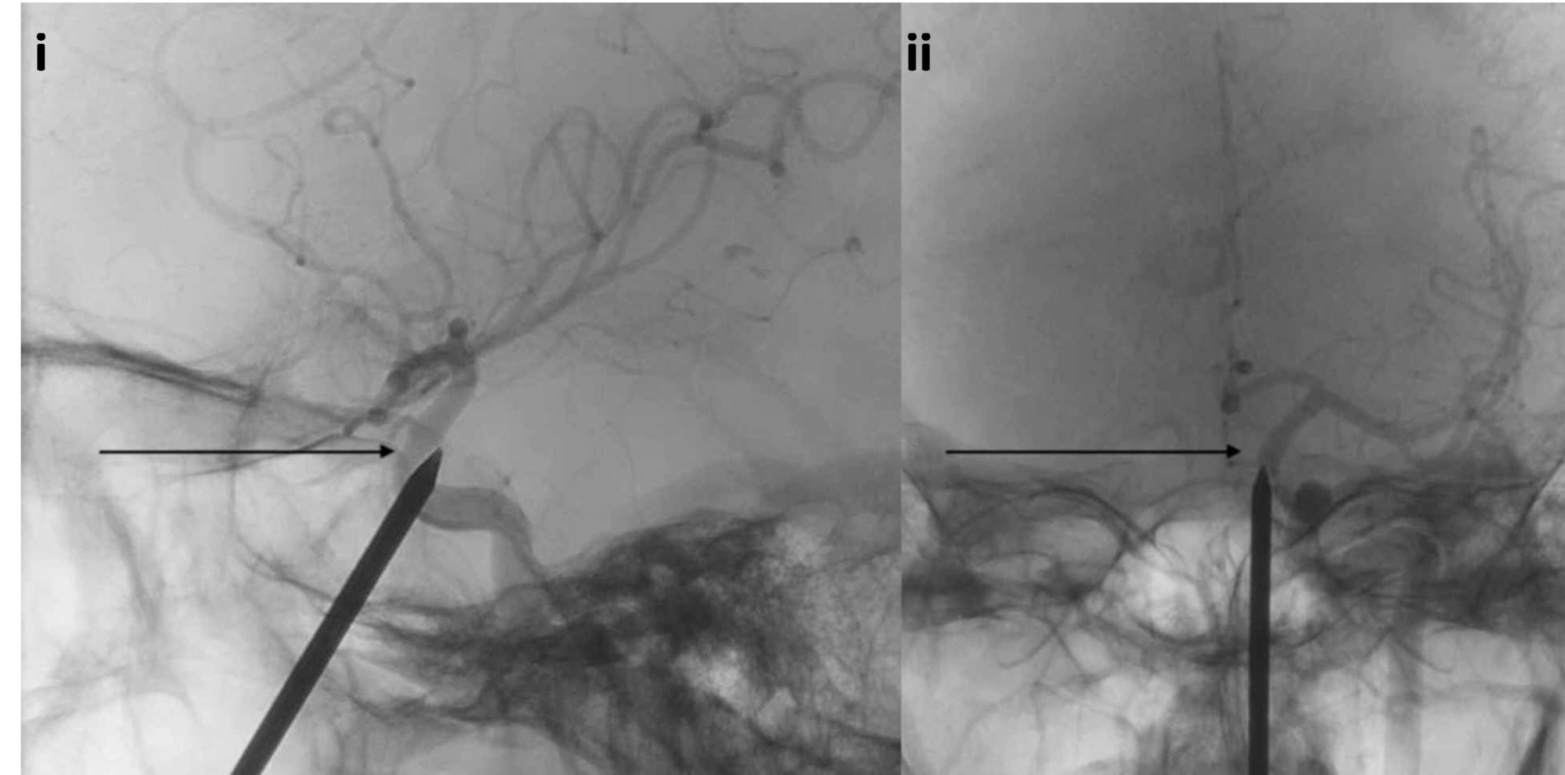
Lateral and AP projection unsubtracted angiogram i) Lateral projection unsubtracted angiogram demonstrating the nail abutting the left cavernous internal carotid artery (black arrow); ii) AP unsubtracted angiogram demonstrating the nail abutting the left cavernous internal carotid artery (black arrow) AP: anterior-posterior

Due to the high likelihood of carotid artery penetration, he was taken to the endovascular suite for the planned removal of the nail with simultaneous DSA and possible intervention for occlusion of internal carotid artery injury. Initial DSA showed the nail abutting the left cavernous internal carotid artery (Figure 15).

**Figure 16:**
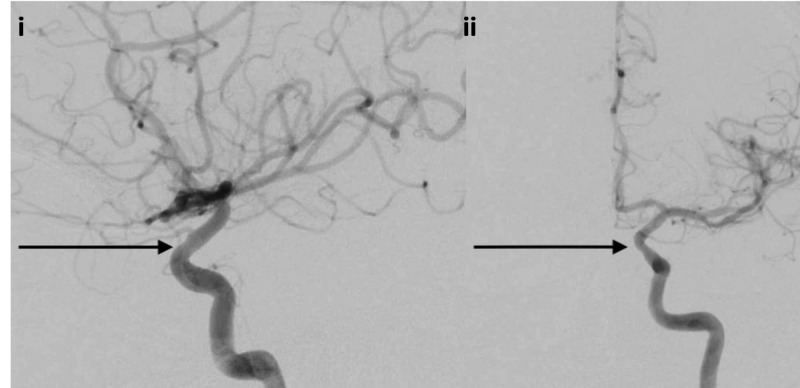
Lateral and AP projection follow-up DSA i) Lateral projection DSA demonstrating mild spasm of the left cavernous internal carotid artery without any evidence of injury (black arrow); ii) AP projection DSA demonstrating mild spasm of the left cavernous internal carotid artery without any evidence of injury (black arrow) DSA: digital subtraction angiography; AP: anterior-posterior

The nail was removed under direct visualization without any evidence of hemorrhage. An immediate follow-up DSA showed mild spasm of the left cavernous internal carotid artery without any evidence of injury, pseudoaneurysm, or dissection (Figure 16).

The patient did have a reported cerebrospinal fluid (CSF) leak post-procedure, which was managed with a lumbar drain. He was discharged home post-injury day 10, neurologically intact and without any further CSF leak.

## Discussion

The type of injuries sustained from low-velocity projectiles differs from medium- and high-velocity projectiles based on the study of ballistics. Kinetic energy is represented by the equation 1/2mv^2^,^ ^where kinetic energy is proportionally related to the mass of the projectile and exponentially related to the velocity of the projectile. As a result, the amount of energy transferred and the resulting degree of tissue injury by a low-velocity penetrating object is comparatively lower. In medium- and high-velocity projectiles, the kinetic energy transferred to the tissue results in radial stretching and cavitation that cause shear forces leading to significant, widespread axonal disruption as well as the endangering of vascular structures [8,9]. Alternatively, low-velocity objects endanger the axons and vascular structures in the penetrating track by direct anatomic disruption without cavitation [8,10,11,12]. Despite the less destructive nature of NPHIs, damage can be incurred when vital structures are involved.

Preoperative management

Initial assessment must include airway, breathing, and circulation to acutely stabilize the patient. Care should be taken to minimize the movement of the foreign body if still retained. None of our cases required manipulation of a retained object to fit through a scanner, but it has been suggested that the proximal portion of an object should be removed when necessary [13]. Once stable, it is suggested that any patient with an intracranial foreign body be transported promptly to a specialized center with access to neurosurgery, neuroendovascular surgery, otolaryngology, and any other subspecialty that could have an influencing role [14,15]. The authors corroborate this suggestion, as indicated by the multidisciplinary approach to treatment in each of our three cases.

Imaging

Head CT is the most effective tool to provide information regarding the involvement of intracranial structures and is, almost, uniformly performed first [7,15,16]. Given that the reported rate of vascular injury in PHI is 5-40%, a CTA is also recommended [7,15,16]. Heightened suspicion for vascular injury should exist with an orbitofacial, paranasal sinus, pterional entry point, intracranial hematoma on initial CT, unexplained subarachnoid hemorrhage, or delayed intraparenchymal hematoma [11]. CTA may be obscured by the metallic artifact of foreign objects, in which case DSA is necessary to delineate the extent of vascular involvement [9-11].

Treatment

First, the patient must be stabilized from a cardiopulmonary perspective. There are occasions where arterial hemorrhage cannot be controlled with packing and this needs to be urgently addressed. Endovascular embolization of arterial hemorrhage in trauma patients has successfully been demonstrated in the pelvis, kidney, mesenteric region, retroperitoneal space, neck, and thigh in addition to controlling epistaxis [17,18]. Once the patient is stabilized, managing a retained foreign body is the next priority. In general, authors have suggested that penetrating head injuries require prompt surgical attention and often removal of an accessible foreign body within 12 hours [9,11,19]. There may be benefit in allowing some time to pass as the foreign object may tamponade any vascular injury permitting clot formation. However, the removal of a foreign body without proper surgical exposure can release this tamponade and result in catastrophic exsanguination and ischemic events to watershed areas [9]. This was likely the scenario in our second case where the nail may have been tamponading the petrous portion of the internal carotid artery. Even though brisk arterial bleeding occurred upon removal, proper surgical exposure prior to nail removal resulted in prompt hemostasis. This second case illustrates that direct visualization of the penetrating object may require an open surgical approach to achieve hemostasis. Furthermore, there should be a high suspicion of vascular injury even in the setting of negative angiographic imaging, and proper preoperative planning with adequate surgical exposure will aid in treating the vascular injury.

By performing the craniotomy before the removal of the nail, we were prepared to control any potential hemorrhage. Previous reports have demonstrated the importance of removing the foreign object in the operating room under direct visualization of the end of the objects while maintaining the patient’s head in a fixed position [14,15]. Our first case also demonstrates the role of endoscopy for direct visualization, although no brisk arterial bleeding was encountered. These cases demonstrate that complete evaluation and surgical management of these objects require a multidisciplinary approach. Formal angiography was utilized prior to the removal of the object in each case. Angiography was also utilized post-removal in cases 1-3, with Onyx embolization and flow-diversion playing a role. In addition to the value an endovascular team has in these NPHIs, an otolaryngology team also has a critical role as well. We have emphasized the significance of direct visualization of both the proximal and distal end of these penetrating objects and this may require an endoscopic approach to accomplish this, as demonstrated in cases 2 and 3. Therefore, to offer a truly comprehensive treatment of these injuries, we propose that a multidisciplinary approach must be employed. We would also like to emphasize the role of endovascular attention in these cases. As reported earlier, there is a 5-40% chance of vascular injury with PHI [7,15,16]. The role of a neuroendovascular provider goes beyond DSA. In our third case, we utilized angiography to look for any hemorrhage following nail removal and were prepared to employ any treatment necessary. In our first case, endovascular treatment included a post-removal angiogram as well as embolization to control bleeding. Endovascular therapy was also used to treat a traumatic pseudoaneurysm with flow-diversion. Therefore, we recommend that endovascular attention be requested in these complex cases. Lastly, debate exists regarding prognosis with NPHI presenting with or without a retained foreign object. Van Dellen and Lipschitz studied knife-induced PHI and suggested that prognosis may be worse when presenting without the offending object, believing its absence was a consequence of the assailant removing it under duress and causing more injury [20]. Taylor and Peter, on the other hand, found a worse prognosis in transcranial stab wound cases with retained blades compared to those without [21]. They attributed the increase in vascular injuries and mortality to deeper penetration, implying a higher likelihood of carotid artery injury. In our case series, every patient was discharged from the hospital with a good neurological outcome. Though our case series is of small sample size, this suggests that if the patient survives the initial injury, hemorrhage is controlled, and secondary insult is prevented, then patients can have excellent neurological outcomes following these injuries.

Postoperative complications

Vascular 

The most common vascular complications among NPHIs are traumatic aneurysm formation and carotid-cavernous fistula [5,7,22,23,24]. Pseudoaneurysms are a particularly common subtype in trauma. They are characterized by disruption of the arterial wall, resulting in the collection of hematoma outside the vessel, contained by a thin layer of adventitia. Due to their lack of neck and vessel wall friability, pseudoaneurysms are not favorable for surgical repair [24]. Sami et al. described a series of pseudoaneurysms treated with flow diversion, which acts to reconstruct flow through the parent vessel and divert flow away from the pseudoaneurysm, thus promoting thrombosis [25]. Four high-risk criteria for developing traumatic aneurysms are passage or bone fragments through areas of crowded vasculature and/or the skull base, large hematoma within the trajectory visible on preoperative, multiple bone fragments scattered in various directions, and a high index of suspicion by the surgeon based on visualization during surgery [22]. Traumatic aneurysms have a 50% mortality and 25.8% stroke rate when untreated [23]. For this reason, a DSA is suggested two-three weeks after the initial injury to assess for aneurysm formation [7]. With our second case, the patient had a flow-diverter placed for a pseudoaneurysm one week after removal of the nail in the operating room. We suggest that patients who satisfy the above criteria should be screened for aneurysm formation once the NPHI is stable.

CSF Leak 

CSF leak is a relatively rare complication of NPHI compared with missile PHIs [5,9,11]. It is postulated that CSF leaks occur frequently in missile PHIs because of a dural tear that cannot be approximated because of the energy and impact caused by the missile [11,12]. We speculate that NPHIs do not cause as much dural and local tissue destruction due to the lower kinetic energy imparted, thus permitting normal healing processes to form a seal. Despite this, our third case, with a penetrance of the sphenoid and cavernous sinuses, did develop a CSF leak that required a lumbar drain. Current guidelines with missile PHIs recommend operative repair of CSF leaks that are not responsive to CSF diversion and we would recommend the same approach for CSF leaks in NPHIs [11].

Infection 

A foreign body in any capacity poses a risk for infection, including severe infections such as brain abscess and meningitis. However, there is no single standard of care regarding the timing of antibiotics, length of the antibiotic regimen, and whether early or prophylactic antibiotics lead to more resistant bacteria strains [11,12]. Recent guidelines have suggested a course of ceftriaxone, metronidazole, or vancomycin for 7-14 days, as such was the case with all three of our patients [11,12]. However, some literature suggests that prophylactic antibiotics should not be employed unless a specific colony has been identified or a specific clinical scenario dictates it [6,7,11]. Ultimately, there is a lack of quality data from randomized controlled trials and there needs to be further research in this area.

Epilepsy 

Seizures are seen in 30-50% of patients following PHI [5,11]. Seizures are particularly prominent in cases with a retained foreign body but have also been attributed to direct injury to the cerebral cortex [5,11,26]. For this reason, our patients received a one-week course of prophylactic seizure medication. None of our three patients had any seizure activity while in the hospital.

## Conclusions

We presented three cases of NPHI and described their unique treatment. Through these cases, we demonstrated the evolving role of endovascular treatment as an adjunct to open surgery. We also discussed an assessment plan with regard to NPHI to stabilize the patient, address uncontrollable bleeding, safely remove a foreign body under direct visualization with endovascular standby, and closely monitor for postoperative complications. Through a multidisciplinary approach, these complex cases have an optimal chance for good outcomes.
